# Elevated fecal calprotectin is associated with gut microbial dysbiosis, altered serum markers and clinical outcomes in older individuals

**DOI:** 10.1038/s41598-024-63893-0

**Published:** 2024-06-12

**Authors:** Sebastian Heinzel, Jenna Jureczek, Veera Kainulainen, Anni I. Nieminen, Ulrike Suenkel, Anna-Katharina von Thaler, Christoph Kaleta, Gerhard W. Eschweiler, Kathrin Brockmann, Velma T. E. Aho, Petri Auvinen, Walter Maetzler, Daniela Berg, Filip Scheperjans

**Affiliations:** 1 Department of Neurology, University Medical Centre Schleswig-Holstein (UKSH), Kiel, Germany; 2 Institute of Medical Informatics and Statistics, University Medical Centre Schleswig-Holstein (UKSH), Kiel, Germany; 3 Human Microbiome Research Program, Faculty of Medicine, University of Helsinki, Helsinki, Finland; 4 Institute for Molecular Medicine Finland, University of Helsinki, Helsinki, Finland; 5 Department of Psychiatry and Psychotherapy, German Center of Mental Health, Tübingen University Hospital, Tübingen, Germany; 6 Institute of Experimental Medicine, Christian-Albrechts-University Kiel and University Medical Center Schleswig-Holstein (UKSH), Kiel, Germany; 7 Geriatric Center, University Hospital Tübingen, Tübingen, Germany; 8 Department of Neurodegeneration, Hertie Institute for Clinical Brain Research, German Center for Neurodegenerative Diseases, University of Tübingen, Tübingen, Germany; 9 Department of Neurology, Helsinki University Hospital, Helsinki, Finland; 10 Department of Clinical Neurosciences (Neurology), University of Helsinki, Helsinki, Finland; 11 Institute of Biotechnology, University of Helsinki, Helsinki, Finland; 12 Department of Neurology, University Medical Centre Schleswig-Holstein, Kiel University, Arnold-Heller-Straße 3, 24105 Kiel, Germany

**Keywords:** Inflammation, Gut microbiota, Calprotectin, Metabolites, Cardiovascular disease, Neurological disease, Clinical microbiology, Chronic inflammation, Biomarkers, Dysbiosis

## Abstract

Fecal calprotectin is an established marker of gut inflammation in inflammatory bowel disease (IBD). Elevated levels of fecal calprotectin as well as gut microbial dysbiosis have also been observed in other clinical conditions. However, systemic and multi-omics alterations linked to elevated fecal calprotectin in older individuals remain unclear. This study comprehensively investigated the relationship between fecal calprotectin levels, gut microbiome composition, serum inflammation and targeted metabolomics markers, and relevant lifestyle and medical data in a large sample of older individuals (n = 735; mean age ± SD: 68.7 ± 6.3) from the TREND cohort study. Low (0–50 μg/g; n = 602), moderate (> 50–100 μg/g; n = 64) and high (> 100 μg/g; n = 62) fecal calprotectin groups were stratified. Several pro-inflammatory gut microbial genera were significantly increased and short-chain fatty acid producing genera were decreased in high vs. low calprotectin groups. In serum, IL-17C, CCL19 and the toxic metabolite indoxyl sulfate were increased in high vs. low fecal calprotectin groups. These changes were partially mediated by the gut microbiota. Moreover, the high fecal calprotectin group showed increased BMI and a higher disease prevalence of heart attack and obesity. Our findings contribute to the understanding of fecal calprotectin as a marker of gut dysbiosis and its broader systemic and clinical implications in older individuals.

## Introduction

Fecal calprotectin is an established marker of gut inflammation, particularly in patients with inflammatory bowel disease (IBD) where it serves as a diagnostic and therapeutic marker of inflammatory disease activity^1^. Calprotectin is a cytosolic protein complex that is constitutively expressed in neutrophils and released when they migrate to the intestinal mucosa during intestinal inflammation^1^. Levels in healthy individuals typically range from 10 to 50 μg per g of stool, whereas in acute inflammatory phases of IBD levels can be > 600 μg/g, with the threshold for IBD diagnosis usually above 100–200 μg/g^1^. However, thresholds differentiating pathological from normal fecal calprotectin ranges lack consensus. While several factors including age, medication, and lifestyle factors may confound fecal calprotectin levels either directly or indirectly^1–3^, evidence regarding systemic and clinical relevance of moderate fecal calprotectin levels is accumulating.

Gut inflammation, even at moderate yet chronic levels, may be involved in various disease processes affecting the central nervous system via the gut-brain axis^4^ and inflammaging^5^. Supporting the systemic relevance of gut inflammation, fecal calprotectin has been shown to be moderately increased to levels between about 50 and 100 μg/g in patients with Parkinson’s disease (PD)^6,7^ and Alzheimer’s disease (AD)^8,9^ compared to controls. Local and systemic pro-inflammatory effects have been partly linked to gut microbial dysbiosis not only in IBD^10^ but also other inflammatory diseases^11^ as well as numerous neurodegenerative^12,13^, metabolic^14^ and cardiovascular diseases (CVD)^15^. For example, gram-negative bacteria like *Fusobacterium* and *Escherichia* which are considered potential pathobionts in IBD^16^, release lipopolysaccharides that can trigger inflammation. In contrast, reduced bacterial abundance in the *Lactobacillaceae*, *Lachnospiraceae* and *Ruminococcaceae* families, which produce short-chain fatty acids (SCFAs) with known anti-inflammatory properties^17,18^, are frequently observed in these diseases. Fecal calprotectin and altered microbiota, microbiome-derived functional pathways, serum inflammation markers and metabolites have been investigated for associations in IBD^10^ and PD^7^, but the systemic and health-related relevance of (moderately) increased fecal calprotectin has not been studied in a large and representative sample of older individuals with a realistic prevalence of age- and lifestyle-related medical conditions.

In the present study we aimed to (I) identify the specific alterations in microbial abundance and diversity at different levels of fecal calprotectin in older individuals, (II) investigate elevated fecal calprotectin for associations with serum inflammation markers and metabolites, (III) explore the role of the gut microbiome as a mediator between fecal calprotectin and serum markers, and (IV) explore lifestyle and clinical manifestations of moderate and high fecal calprotectin including health-related quality of life, (cardio)vascular, metabolic and inflammatory diseases, (prodromal) PD/AD, and depression.

## Results

### Fecal calprotectin and characteristics of the study population

**Figure 1:**
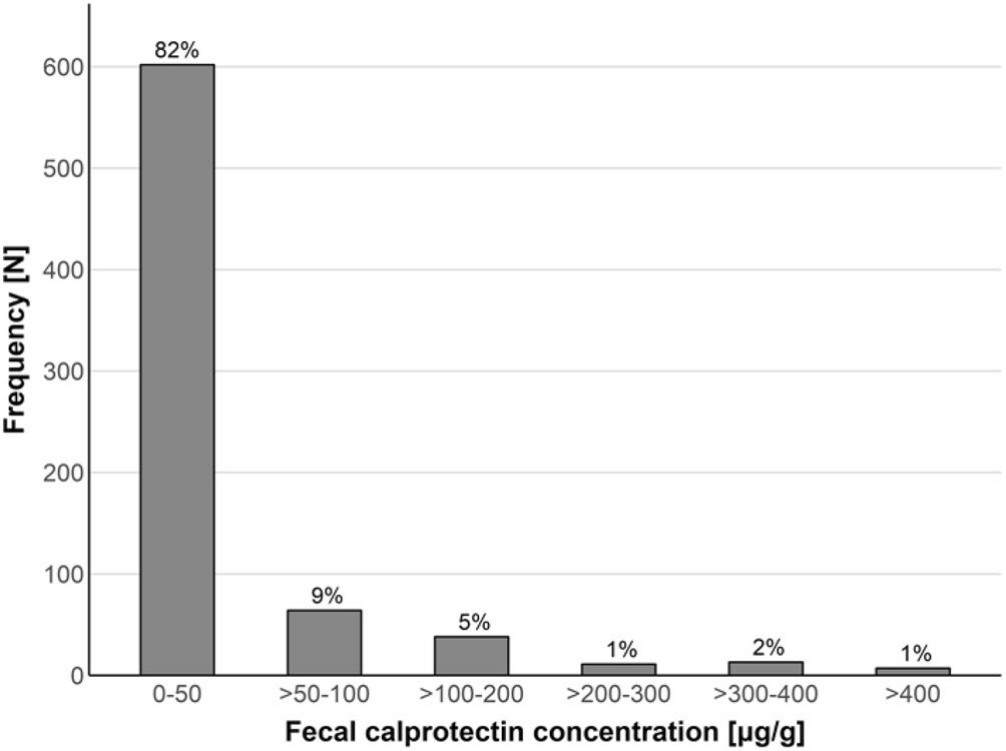
Distribution of fecal calprotectin for different concentration intervals in the study population of older individuals (n = 735).

Of the participants (n = 728 after exclusion of 7 fecal calprotectin outliers, mean age ± SD, 68.7 ± 6.3, range: 53–86) of the TREND study, 8.5% (n = 62) showed high fecal calprotectin levels exceeding 100 μg/g. The vast majority (91.5%, n = 666) showed levels at or below 100 μg/g (Fig. 1). Overall, fecal calprotectin levels had a mean ± SD of 35.0 ± 61.5 μg/g, a median of 14 μg/g, and a range of 0 to 382 μg/g. The descriptive statistics and potential confounders of fecal calprotectin differing between stratified groups are shown in Table 1. Increasing calprotectin was associated with increasing body mass index (BMI) and age as well as with more frequent intake of proton-pump inhibitors (PPIs) and nonsteroidal anti-inflammatory drugs (NSAIDs). Moreover, lower preference for dark bread was observed for the high vs. low calprotectin groups. The moderate calprotectin group had a more frequent intake of a variety of medications compared to the low calprotectin group. To account for distinct profiles of differences in potential confounders for each pair-wise group comparison, the variables showing significance (with p < 0.05 in logistic regressions accounting for age, sex and BMI) for each comparison were selected as covariates for subsequent analyses (Table S1).

**Table 1:** Descriptive statistics of variables associated with elevated fecal calprotectin levels and testing of high or moderate vs. low fecal calprotectin groups for differences in these variables using Wilcoxon or Fisher’s exact tests.

	Low (≤ 50 μg/g)	Moderate (> 50–100 μg/g)	High (> 100 μg/g)	Moderate vs low	High vs low
	Mean (SD) or frequency (%)			p-value	
Demographic/physiological					
Body mass index [kg/m^2^]^1,2^	26.4 (4.4)	27.6 (4.1)	28.8 (4.5)	**0.013**	** < 0.001**
Age [years]^1,2^	68.3 (6.1)	70.6 (7)	70.2 (6.9)	**0.009**	**0.033**
Female sex	297/602 (49%)	25/64 (39%)	25/62 (40%)	0.147	0.185
Health condition					
Heart attack^1^	21/602 (3%)	4/64 (6%)	9/62 (15%)	0.288	**0.001**
Hypertension	263/599 (44%)	34/64 (53%)	37/62 (60%)	0.186	**0.022**
Arthritis^2^	115/602 (19%)	24/64 (38%)	18/62 (29%)	**0.001**	0.068
Stroke	31/602 (5%)	6/64 (9%)	7/62 (11%)	0.155	0.076
Medication					
Proton-pump inhibitors^1,2^	46/598 (8%)	24/64 (38%)	21/61 (34%)	** < 0.001**	** < 0.001**
NSAID^1^	133/602 (22%)	23/64 (36%)	26/62 (42%)	**0.019**	**0.001**
Any antihypertensive drugs	266/598 (44%)	37/64 (58%)	38/61 (62%)	**0.048**	**0.01**
Any probiotics	51/575 (9%)	3/64 (5%)	1/59 (2%)	0.345	0.076
Urate lowering drugs	22/598 (4%)	7/64 (11%)	5/61 (8%)	**0.016**	0.094
Antidiabetic drugs	35/598 (6%)	4/64 (6%)	7/61 (11%)	0.783	0.097
Dyslipidemia drugs	120/598 (20%)	19/64 (30%)	18/61 (30%)	0.077	0.098
Tricyclic drugs^2^	12/597 (2%)	5/64 (8%)	3/61 (5%)	**0.018**	0.155
Class 3 antiarrhythmic drugs^2^	1/597 (0%)	2/64 (3%)	1/61 (2%)	**0.026**	0.177
Beta2 mimetic drugs^2^	23/598 (4%)	8/64 (12%)	0/61 (0%)	**0.006**	0.258
Cortisone drugs^2^	32/598 (5%)	9/64 (14%)	1/61 (2%)	**0.012**	0.351
SSRI^2^	16/598 (3%)	6/64 (9%)	2/61 (3%)	**0.014**	0.679
Any antidepressant drugs^2^	53/598 (9%)	12/64 (19%)	5/61 (8%)	**0.024**	1
NDRI^2^	3/598 (1%)	3/64 (5%)	0/61 (0%)	**0.014**	1
Lifestyle^a^					
Dark bread preferred^1^	283/596 (47%)	24/64 (38%)	15/61 (25%)	0.147	**0.001**
Dairy consumption (milk and cheese)	1.7 (0.9)	1.9 (1)	1.5 (0.8)	0.131	0.058
Meat consumption (incl. cold cuts)	1.3 (0.7)	1.4 (0.7)	1.5 (0.6)	0.796	0.059
Dietary supplements^2^	154/598 (26%)	27/64 (42%)	9/61 (15%)	**0.007**	0.062
Physically inactive^2^	103/600 (17%)	22/64 (34%)	16/62 (26%)	**0.002**	0.116

^1^Variable retained as covariate in subsequent analyses of High vs Low calprotectin group comparisons.

^2^Variable retained as covariate in subsequent analyses of Moderate vs Low calprotectin group comparisons.

^a^Definitions and units are given in the Supporting material (Variable list).

Significant values are in bold.

### Gut microbial differences

**Figure 2:**
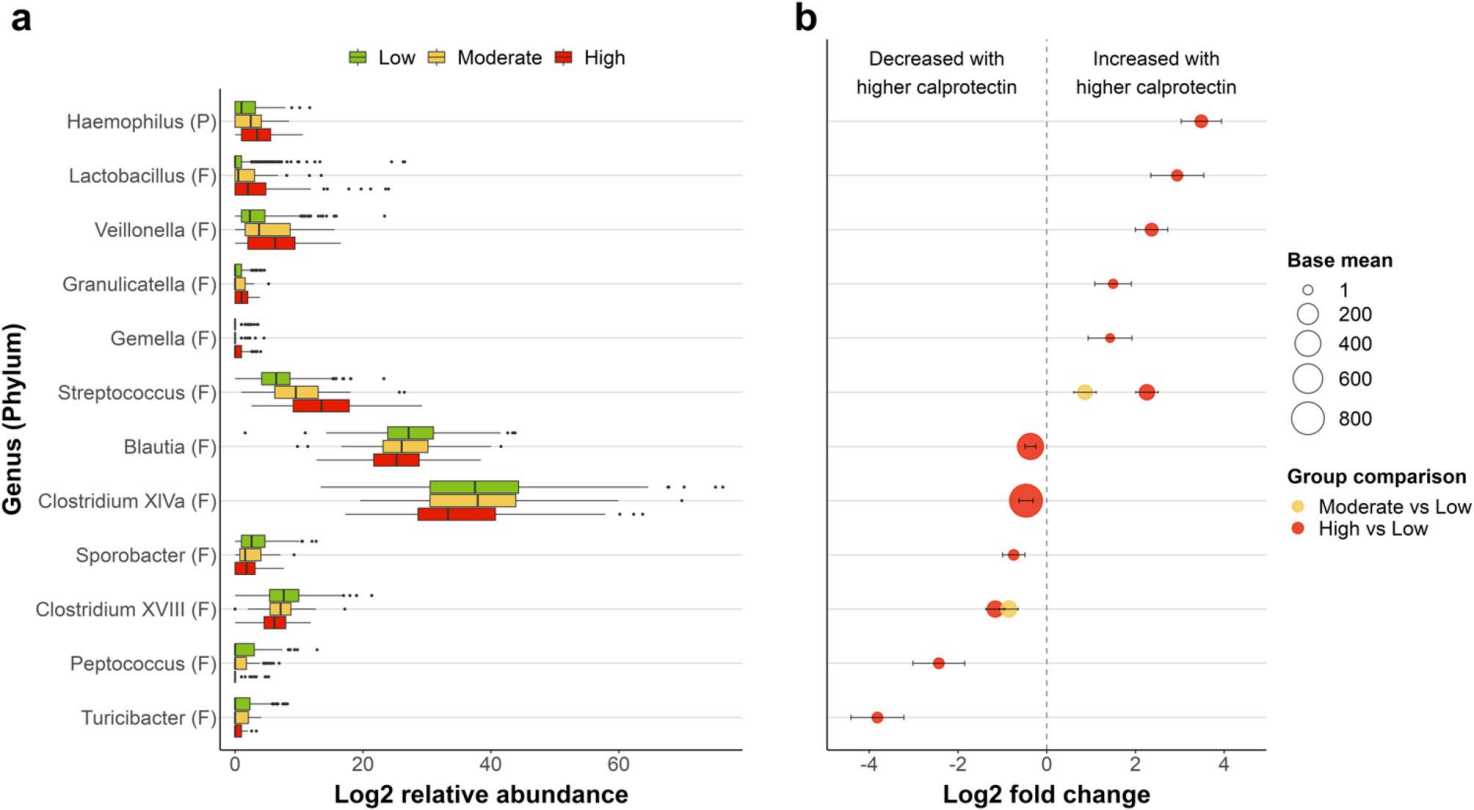
Genus-level differential abundance analysis. (**a**) Group-wise boxplots of the relative abundance (log2) of genera that show significant differences in group contrasts. (**b**) Significant (FDR-corrected, covariate-adjusted) findings of differential abundance of gut microbial genera for comparisons between moderate (> 50–100 μg/g) or high (> 100 μg/g) vs. low (≤ 50 μg/g) fecal calprotectin groups. The size of the markers corresponds to the base mean value (the mean of normalized counts of all samples) and the markers are color-coded based on group comparison. Bars indicate the standard error of log2-fold change. The phylum of the genera is indicated in brackets. P *Proteobacteria*, F *Firmicutes*.

Individuals with high fecal calprotectin levels showed significant differences (p < 0.05; FDR-adjusted, accounting for covariates) in gut microbial abundance of 12 genera compared to groups with low calprotectin, including an increased abundance of gram-negative *Haemophilus* and *Veillonella* and a decreased abundance of SCFA-producing genera, e.g. *Clostridium* groups*, Blautia* and *Turicibacter* (Fig. 2, Table S2a–c). A significant increase in *Streptococcus* and decrease in *Clostridium XVIII* was observed between both moderate vs. low and high vs. low fecal calprotectin groups.

Among covariates, constipation and physical inactivity especially showed numerous significant results in the differential abundance analyses (Table S2a–c). Largely consistent results were observed for the differential abundance on the taxonomic level of families (Fig. S1). Fecal calprotectin as a continuous variable also showed largely consistent results with the group comparisons in differential abundance analyses (Fig. S2).

α-diversity (inverse Simpson and Shannon index) and β-diversity (Bray–Curtis distances) showed no significant differences between high or moderate vs. low fecal calprotectin groups (p > 0.1; Tables S4, S5).

### Functional pathway differences

In total, 388 functional pathways were identified via PICRUSt2, with 26 involving SCFA production. In the differential abundance analysis of high vs. low fecal calprotectin groups, 11 pathways showed a nominally significant (p < 0.05, no FDR-correction, accounting for covariates) difference including three SCFA pathways (Table S3). Among the pathways related to the fermentation to SCFAs, heterolactic fermentation (MetaCyc pathway: P122-PWY), and Bifidobacterium shunt (P124-PWY) were decreased, whereas hexitol fermentation to lactate, formate, ethanol, and acetate (P461-PWY) was increased in high vs. low fecal calprotectin groups. Moreover, relative decreases were observed for pathways related to amino acid biosynthesis (ARGSYNBSUB-PWY), carbohydrate biosynthesis (COLANSYN-PWY), nucleoside and nucleotide biosynthesis (PWY-7187, PWY0-166) and sugar degradation (FUCCAT-PWY, FUC-RHAMCAT-PWY), whereas carboxylic acid degradation (GLUCARDEG-PWY), and glycolysis (P341-PWY) were increased.

### Serum markers

**Figure 3:**
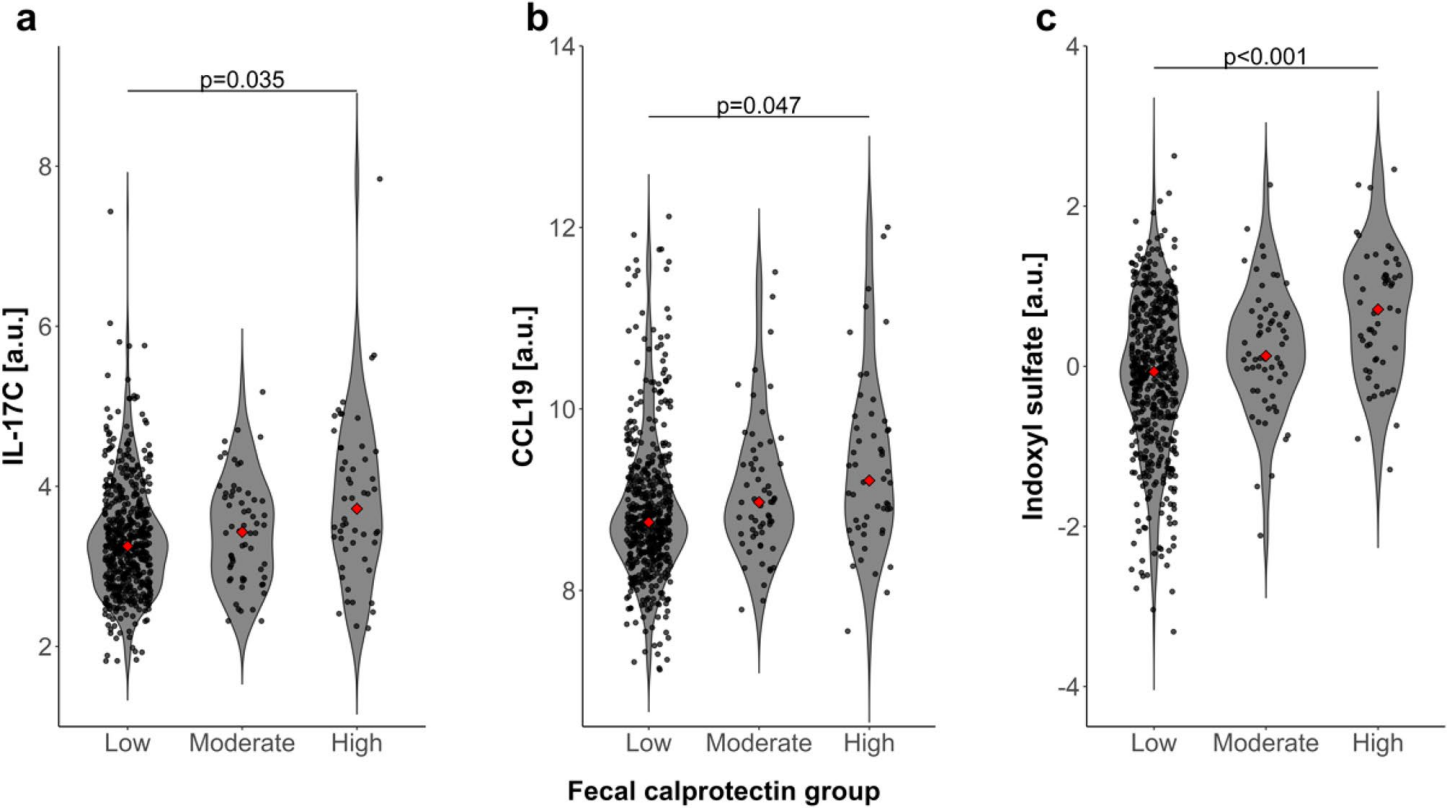
Serum markers with significant differences between fecal calprotectin groups. (**a**) IL-17C, (**b**) CCL19 and (**c**) indoxyl sulfate were identified through multiple regression with potential confounders of fecal calprotectin included as covariates. The p-values have been FDR corrected for the number of 92 inflammation markers and 135 metabolites. Serum marker values were normalized and reported with arbitrary units. Red points indicate median values.

Among serum inflammation markers, IL-17C (p = 0.035, FDR-adjusted, accounting for covariates) and CCL19 (p = 0.047, FDR-adjusted, accounting for covariates) showed significantly increased levels in the high compared to the low fecal calprotectin group (Fig. 3a,b). Among serum metabolites, indoxyl sulfate (p < 0.001, FDR-adjusted, accounting for covariates) was significantly increased in the high vs. low fecal calprotectin group comparison (Fig. 3c). None of the other serum inflammation markers or metabolites showed significant differences between groups after accounting for multiple testing of markers. Three SCFAs—acetate, butyrate, and propionate—were among the metabolites measured and tested for fecal calprotectin group differences. However, none showed significant differences in any group comparison (uncorrected p > 0.1). Results of inflammation markers and metabolites showing group differences with uncorrected p < 0.05 are shown in Supplementary Tables S6 and S7.

### Mediation analysis

The associations between fecal calprotectin and IL-17C, CCL19 and indoxyl sulfate were tested for mediation effects of the gut microbiome. In the high vs. low fecal calprotectin groups, indoxyl sulfate showed a global mediation effect (p < 0.001, FDR corrected, accounting for covariates) and five genera-specific effects (Table 2). There was no mediation effect observed for either IL-17C or CCL19, nor for the moderate vs. low group comparison.

**Table 2:** Gut microbiome as a mediator of the associations between fecal calprotectin and serum marker levels.

Calprotectin groups	Serum marker	Global* p-value	Significant mediators**	Microbial mediation effects			
				Exposure (calprotectin group)		Exposure-adjusted outcome (serum marker)	
				β coefficient	p-value	β coefficient	p-value
High vs low	Indoxyl sulfate	< 0.001	*Haemophilus*	0.254	< 0.001	− 0.045	< 0.001
			*Veillonella*	0.173	< 0.001	− 0.029	0.002
			*Blautia*	− 0.413	< 0.001	0.525	0.003
			*Clostridium XlVa*	− 0.704	< 0.001	− 0.624	0.003
			*Turicibacter*	− 0.041	< 0.001	− 0.049	< 0.001
	IL-17C	0.721	–	–	–	–	–
	CCL19	0.879	–	–	–	–	–

*Global refers to the global mediation effect of all of the 12 genera showing significant high vs low fecal calprotectin effects in the differential abundance analysis.

**Inverse regression that regresses the microbiome data at each taxon on the exposure and the exposure-adjusted outcome. Separate mediation analysis results of the mediation effects are shown for individual taxa among the 12 genera.

### Health-related and clinical variables

Finally, we explored potential links between fecal calprotectin groups and clinical variables, including health-related quality of life scores, (cardio)vascular, metabolic and inflammatory diseases, and (prodromal) PD, (prodromal) AD and depression (Table 3). The high calprotectin group contained more individuals with a positive history of heart attack compared to the low group. Moreover, BMI and prevalence of obesity markedly increased with higher fecal calprotectin. Health-related quality of life was reduced in individuals with moderate but not those with high calprotectin levels compared to the low calprotectin group. Motor deficits indicating subthreshold parkinsonism were more frequently observed for the moderate compared to the low fecal calprotectin group, as however was the prevalence of arthritis which could confound motor deficits. These differences were not observed in the high vs. low group comparison. Scores for cognitive performance and depression did not differ significantly between calprotectin groups. Sample sizes of individuals with prodromal PD probabilities of > 50%^19^, mild cognitive impairment (MCI), incident PD and AD (i.e. newly diagnosed disease two or four years after the stool sampling), and prevalent PD and AD cases at time of sampling were generally very small, and no differences in the prevalence were observed between fecal calprotectin groups.

**Table 3:** Differences in clinical variables between high/moderate and low fecal calprotectin groups in multiple/logistic regression with age, sex and BMI.

	Low (≤ 50 μg/g)	Moderate (> 50–100 μg/g)	High (> 100 μg/g)	Moderate vs low	High vs low
	Mean (SD) or frequency (%)			p-value	
Subjective health status					
Health-related QOL score	78.1 (14.1)	72.1 (18)	75.4 (12.5)	**0.009**	0.229
(Cardio)vascular disease					
Heart attack	21/602 (3%)	4/64 (6%)	9/62 (15%)	0.504	** < 0.001**
Stroke	31/602 (5%)	6/64 (9%)	7/62 (11%)	0.204	0.158
Heart failure	46/602 (8%)	10/64 (16%)	7/62 (11%)	0.104	0.675
Hypertension	263/599 (44%)	34/64 (53%)	37/62 (60%)	0.679	0.265
Metabolic disease					
Body mass index [kg/m^2^]	26.4 (4.4)	27.6 (4.1)	28.8 (4.5)	**0.042**	** < 0.001**
Obesity	103/599 (17%)	15/63 (24%)	22/62 (35%)	0.17	**0.001**
Diabetes type II	47/602 (8%)	5/64 (8%)	8/62 (13%)	0.668	0.77
High cholesterol	254/598 (42%)	27/64 (42%)	23/61 (38%)	0.92	0.343
Hyperlipidemia	358/602 (59%)	42/64 (66%)	43/62 (69%)	0.6	0.285
Inflammatory disease					
Arthritis	115/602 (19%)	24/64 (38%)	18/62 (29%)	**0.003**	0.127
Irritable bowel syndrome	27/596 (5%)	4/64 (6%)	5/61 (8%)	0.341	0.134
(Prodromal) Parkinson’s disease					
MDS-UPDRS-III motor score	1.6 (3.4)	3 (5.3)	2.5 (3.5)	**0.044**	0.09
Probability of prodromal PD [%]	3.9 (12.3)	8 (20.4)	6.6 (16.1)	0.163	0.363
Incident Parkinson’s disease	9/602 (1%)	1/64 (2%)	0/62 (0%)	0.702	0.993
Parkinson’s disease	8/602 (1%)	0/64 (0%)	1/62 (2%)	0.99	0.933
(Prodromal) Alzheimer’s disease					
CERAD sum score	85.2 (7.9)	82.9 (9.3)	83.3 (9.2)	0.441	0.781
Mild cognitive impairment	27/602 (4%)	1/64 (2%)	6/62 (10%)	0.244	0.177
Incident Alzheimer’s disease	8/602 (1%)	0/64 (0%)	0/62 (0%)	0.994	0.994
Alzheimer’s disease	3/602 (0%)	1/64 (2%)	0/62 (0%)	0.499	0.997
Depression					
Depressivity (BDI-II sum score)	6.5 (6.6)	7.7 (7)	6 (5.1)	0.146	0.621
Depression	197/602 (33%)	20/64 (31%)	17/62 (27%)	0.881	0.525

Significant values are in bold.

Sensitivity analyses showed some differences compared to these results when including seven individuals with extreme fecal calprotectin levels (> 400 μg/g) that were excluded due to the possibility of acute infection and/or undiagnosed IBD. Among clinical variables, significantly more prevalent irritable bowel syndrome, and regarding microbiome data, decreased abundance in three additional taxa (*Eubacterium*, *Clostridium_sensu_stricto* and *Holdemania*) in low vs. high/extreme calprotectin groups were observed. While IL-17C and CCL19 were no longer significantly different (p = 0.060 and p = 0.166) when including individuals with extreme fecal calprotectin levels, the finding of decreased indoxyl sulfate remained unchanged (p < 0.001).

## Discussion

The present study is the first to comprehensively investigate a large cohort of older individuals for associations between fecal calprotectin levels and gut microbiome composition as well as serum inflammation and metabolite markers and a wide range of clinical variables. While accounting for potential confounders, we identified several specific gut microbial differences, in particular between individuals with high compared those with low fecal calprotectin, that are consistent with previous findings of gut microbial dysbiosis in IBD^20^. Genera that can induce inflammation or are adapted to pro-inflammatory environments were increased, and key SCFA-producing bacteria were decreased in high vs. low calprotectin groups. In serum, significant increases in IL-17C, CCL19 and indoxyl sulfate in the high vs. low fecal calprotectin group suggest systemic relevance of high fecal calprotectin. The increase of indoxyl sulfate in the high vs. low fecal calprotectin was partially mediated by the gut microbiota. Moreover, we replicated and extended previous findings of associations between increased fecal calprotectin levels and medications such as PPI and NSAIDs, BMI, and diet/lifestyle factors. Elevated fecal calprotectin levels may have clinical and potentially pathomechanistic relevance for health-related quality of life, cardiovascular and inflammatory diseases as well as for obesity. Fecal calprotectin showed no association with depression, cognitive performance and (prodromal) neurodegenerative diseases.

Changes in microbial composition associated with elevated fecal calprotectin levels could be attributed to various lifestyle, aging, disease and physiological gut-related and/or systemic factors with partly complex, direct and indirect interrelations^21^. Microbial adaptation to pro-inflammatory environments can favor the proliferation of certain bacterial genera^21^, which might have partly contributed to the altered abundances of genera observed in groups with elevated calprotectin. For instance, higher levels of oxygen in the inflamed gut can permit aerobic respiration by Enterobacteriaceae, while inhibiting growth of obligate anaerobes *Bacteroidia* and SCFA-producing *Clostridia*^21^*.* Additionally, inflammation and elevated fecal calprotectin levels might, through interaction with fat-rich Western diets, be triggered by gram-negative bacteria containing pro-inflammatory lipopolysaccharides as membrane components^22^. Bacteria of the genus *Haemophilus* (phylum *Proteobacteria*), which were increased in high vs. low calprotectin groups, are gram-negative, and several strains may be pro-inflammatory. These bacteria have been reported to be increased in IBD patients, in particular during inflammatory flares^23^, and have been associated with more severe disease progression in pediatric ulcerative colitis patients^24^. *Streptococcus* has also been shown to be increased in abundance in IBD^11^ and as a risk factor for post-operative IBD relapse^16^. Furthermore, *Streptococcus* has been reported to be increased in abundance in (sub)clinical atherosclerotic cardiovascular disease^15,25^, AD^12^, PD^13^, and obesity^14^. While some species of *Lactobacillus* are considered as probiotics that promote anti-inflammatory responses and gut barrier maintenance^26^, *Lactobacillus* has also been shown to be increased in abundance in atherosclerotic cardiovascular disease^15^ as well as in AD^27^ and PD^13^, yet little evidence points to alterations of *Lactobacillus* in IBD. The genus does comprise both species with beneficial probiotic effects^28^ as well as opportunistic pathogens associated with, e.g. endocarditis, bacteremia and pleuropneumonia^29^, which complicates the interpretation of the role of *Lactobacillus* for (gut) health*.* Moreover, consistent with the increased abundance in the high vs. low calprotectin groups, *Veillonella* (*parvula*) has been shown to be enriched in Crohn’s disease^30^, and is considered a commensal found in the oral and intestinal tract, previously found also as pathogen in cases of meningitis^31^ and periodontitis^32^. However, the intake of drugs such as PPIs has been shown to be associated with increases in several of these taxa, and while the intake of a wide range of specific medications was accounted for as covariates in the analyses of fecal calprotectin group comparisons, it is possible that our findings of differential microbial abundance might in part still be related to medication^33^.

The reduction in the abundance of several SCFA-producing genera, e.g. *Clostridium* groups*, Blautia* and *Turicibacter*, in groups with elevated fecal calprotectin aligns with many findings in IBD^16,20^, as well as PD^13^ and cardiovascular diseases^34^. A reduction in these SCFA-producing genera may be pivotal for several disease mechanisms as SCFAs can protect against pathogens, regulate metabolic, endocrine, and immune functions, and influence drug metabolism and absorption^18^. Thereby, gut microbiota (and gut inflammation) play a systemic role of multidirectional communication and signaling of inflammation beyond the gastrointestinal tract along several proposed axes involving the gut, liver, heart and brain^4,34,35^. We did not observe significant differences in serum SCFAs between calprotectin groups, however SCFAs and relative reductions thereof may be difficult to detect in serum due to their systemic metabolization.

The analyses of the abundance of functional pathways in the stool samples indicated a decrease in the production of acetate, lactate and propionate in the high vs. low calprotectin groups. Acetate is the primary energy source of gut endothelial cells and can be transformed into the SCFA butyrate, which has many of aforementioned systemic functions^18^. Lactate can also be fermented to butyrate but has also been shown to accelerate epithelial development^36^, which could be relatively reduced in the high calprotectin group. Propionate has been shown to mitigate the effects of LPS on the blood–brain barrier and has also been associated with a protective effect against cardiovascular diseases^37,38^. However, the results were partially inconsistent as one pathway involved in the production of acetate and lactate was increased in the high calprotectin group. This underscores the intricate nature of the gut microbiota and their diverse functions. Diversity measures of microbial composition indicated by intra-individual α-diversity and inter-individual differences in microbial composition (β-diversity) did not show significant differences between fecal calprotectin groups when accounting for covariates. Previous evidence suggests a reduction in α-diversity in IBD and differences in microbial composition between IBD patients and controls^16^. However, alterations in microbial diversity and functional pathways in individuals with elevated calprotectin may co-occur with the presence of several potential confounders and/or causal factors of gut inflammation, complicating the disentanglement of their independent effects.

The serum inflammation marker IL-17C belongs to the IL-17 cytokine family and is produced by epithelial cells rather than immune cells. It acts as a rapid local autocrine response to epithelial injury promoting anti-microbial protective responses and intestinal barrier maintenance^39^. Thus, the increase in IL-17C in the high vs. low fecal calprotectin group is plausible. Furthermore, increases in IL-17C serum levels, IL-17C mRNA in inflamed colonic lesions, and heightened IL-17C staining in active IBD colonic sections in IBD patients suggest a pathological involvement of the IL-17C pathway in IBD^40^. CCL19 is a chemokine which has a crucial role in regulating the recruitment and enhancing the response of inflammatory T-cells as well as guiding immune cells including lymphocytes and dendritic cells to sites of inflammation^41^. Our finding may indicate an amplified immune and barrier maintenance response in the high calprotectin group.

Among serum metabolites, indoxyl sulfate was significantly increased in the high vs. low calprotectin group comparison. Mediation analyses showed that the associations between fecal calprotectin and indoxyl sulfate are mediated by the microbiome including both taxa with increased as well as those with decreased abundance in the high vs. low fecal calprotectin groups. Thus, thus indoxyl sulfate levels and their associations with fecal calprotectin appear to depend on various specific features of microbial composition. Indoxyl sulfate is a metabolite of dietary tryptophan. Tryptophan is converted to indole by resident gut microbes and further metabolized by the liver to produce indoxyl sulfate^42^, which is considered a cardiotoxin^43^ and uremic toxin^44^. Indoxyl sulfate can suppress tight junction-related genes and inhibit mitophagy in intestinal cells, leading to intestinal barrier damage^45^. It has also been linked to the production of inflammatory cytokines, resulting in cardiac fibrosis and hypertrophy in a rat model^46^, as well as increased CVD events in humans^42^. Moreover, indoxyl sulfate in cerebrospinal fluid has been shown to be elevated in patients with multiple sclerosis, to positively correlate with markers of neurodegeneration, and to be reduced after disease-modifying (dimethyl fumarate) therapy^47^. Diet plays a crucial role in indoxyl sulfate production, with increased indoxyl sulfate levels in diets with high protein and lower levels in vegetarian diets^42^. Nevertheless, fecal calprotectin groups did not differ significantly in these dietary variables after accounting for age, sex and BMI suggesting that diet did not confound indoxyl sulfate differences between groups. In the high vs. low calprotectin group comparison, we observed an increase in both indoxyl sulfate levels and a higher rate of heart attacks in the medical history. While high fecal calprotectin has so far not been associated with heart attacks, studies have linked high serum calprotectin levels to CVD^48^. However, stool samples and thus fecal calprotectin might not have been sufficiently investigated, yet.

Groups with moderate fecal calprotectin levels exhibited decreased health-related quality of life. Additionally, the high calprotectin group demonstrated an elevated prevalence of cardiovascular disease, obesity, and higher BMI compared to the low calprotectin group. The specific microbial alterations, increases in indoxyl sulfate and the more prevalent history of heart attacks observed in the high vs. low calprotectin groups suggest crucial interdependencies between gut inflammation and cardiovascular diseases. Conversely, clinical measures of the gut-brain axis including depression and cognitive performance and (prodromal) neurodegenerative diseases did not show any significant differences between fecal calprotectin groups. However, the number of (prodromal/incident) PD and AD cases was very small in the present study and the potential role of gut inflammation for neurodegenerative diseases requires further investigation.

In our analysis of potential confounders in high vs. low fecal calprotectin groups, we replicated previously reported associations between PPI intake and increased fecal calprotectin levels^9^. PPI can, increase pH levels, and thereby, extend the half-life of calprotectin^3^ and facilitate the translocation of pathogens to the gut by reducing stomach acids, potentially leading to increased inflammation. Thus, while PPI intake could represent a confounder of fecal calprotectin it may also constitute a causal factor underlying gut inflammation. NSAIDs can cause mucosal bleeding^3^ and impair the intestinal barrier^49^, and might thereby contribute to gut inflammation and elevated fecal calprotectin levels. Our findings of strongly increased BMI and obesity with higher fecal calprotectin levels are both consistent with the correlation of serum calprotectin and BMI in healthy children^50^, as well as obesity as a risk factor of Crohn’s disease (but not ulcerative colitis)^51,52^. BMI may influence fecal calprotectin levels both indirectly, e.g., via pro-inflammatory diets and sedentary behavior, and directly via (systemic) inflammatory processes, similarly to what is observed in metabolic diseases and CVD^53.^ The increased preference of dark (fiber-rich) over white bread in the group with low compared to high calprotectin suggests this dietary choice reduces inflammation. Possibly, this association involves anti-inflammatory SCFAs made from fermentation of dietary fiber and resistant starch by gut bacteria^54^. While our sample did not include clinically diagnosed IBD cases, and we excluded seven individuals with extreme fecal calprotectin levels, the high calprotectin group may still contain individuals with undiagnosed IBD. Importantly, the potential confounders showed a multitude of (independent) associations with altered microbial genera and microbiome-derived functional pathways, as well as serum inflammation and metabolite markers. In part, effects of these covariates are difficult to disentangle from effects of fecal calprotectin, however several findings were still robust against rigorous accounting for these covariates in pair-wise calprotectin group comparisons. Interestingly, comparisons of the moderate vs. low fecal calprotectin groups showed a several medications to be more frequently used in the moderate group, suggesting a more complex medication regime with possible impact on microbial composition in this group.

Strengths of this study include a large sample size, standardized biomarker measurements conducted in a single laboratory, and comprehensive lifestyle and clinical assessments allowing for the investigation and accounting for numerous potential confounders of fecal calprotectin levels. However, there are several limitations to consider. First, fecal calprotectin has high sensitivity but low specificity, as it can be significantly elevated during infections^1^ which were not assessed in the TREND study. However, we did exclude individuals with extreme fecal calprotectin values as those might have had an acute infection and/or undiagnosed IBD. Our rationale for excluding these individuals was confirmed given that significantly more prevalent irritable bowel syndrome and differences in inflammation marker findings in the comparison of high/extreme vs. low calprotectin groups were observed when including these fecal calprotectin extremes. Second, while we investigated a large and realistic sample of older individuals, the subgroups with specific clinical diagnoses were smaller in comparison to some case–control studies, potentially affecting the statistical power, particularly for examining associations between (prodromal) neurodegenerative diseases and fecal calprotectin. Third, we only analyzed one timepoint thus intraindividual variance of fecal calprotectin and chronic elevation of fecal calprotectin could not be investigated.

In conclusion, this study unveiled intricate associations between fecal calprotectin levels and microbial and serum marker alterations in older individuals. Notably, individuals with high fecal calprotectin levels exhibited marked gut microbial dysbiosis reminiscent of changes observed in patients with IBD, CVD and neurodegenerative diseases. Elevated serum IL-17C and CCL19 levels suggest increased anti-microbial protective responses and intestinal barrier maintenance in the high calprotectin group. Increased (cardio)toxic serum indoxyl sulfate, partly mediated by the gut microbiome, and a higher prevalence of heart attack in the high fecal calprotectin group suggest a link between gut inflammation and cardiovascular health. Moreover, we confirmed several previously established links between fecal calprotectin and the use of PPIs and NSAIDs, while also identifying BMI as a relevant covariate. The present findings contribute to the understanding of the role of fecal calprotectin in gut microbial dysbiosis, and its systemic and clinical implications in older individuals.

## Patients and methods

### Study design and population

We analyzed data of the prospective Tübingen Evaluation of Risk Factors for Early Detection of Neurodegeneration (TREND) study (www.trend-studie.de/english). The TREND cohort of older individuals (aged 50+ years at baseline; total n = 1201) has been studied longitudinally in six waves from 04/2009 to 03/2020 every 2 years by multimodal and multidisciplinary data acquisition. The participants were recruited from the general population, but the cohort has been partly enriched with individuals exhibiting established PD and AD risk factors, including life-time depression, olfactory loss, and/or possible REM-sleep behavior disorder. All subjects gave written informed consent to participate in the study.

In the present study, all biosamples and cohort data analyzed were collected at the fourth wave (follow-up 3; 2015/2016) of the TREND study. Stool samples were available for 745 participants. For 10 samples fecal calprotectin measurements were missing, and 7 samples were excluded as outliers with extreme fecal calprotectin values (> 400 μg/g), thus data of n = 728 older individuals were considered in the cross-sectional analyses^55^.

### Ethics declaration

The study was approved by the local ethics committee (Medical Faculty, University of Tübingen; 444/2019BO2), and complies with the standards of the declaration of Helsinki in its latest version. Written informed consent was obtained from all participants.

### Biological variables

Stool was sampled using collection tubes with a DNA stabilizer for the microbiome analyses, and without DNA stabilizer for the fecal calprotectin measurements (PSP Spin Stool DNA Plus Kit; STRATEC Molecular, Birkenfeld, Germany), which were provided using postal services and frozen and stored at − 80 °C immediately upon arrival. Serum was stored at − 80 °C within 60 min after collection and analyzed without any previous thaw–freeze cycle. Fecal calprotectin was quantified as μg per g feces using standard procedures in an established and validated quantitative immunofluorometric assay (EliA Calprotectin 2 test; ThermoFischer Scientific). The assay had a detection threshold of 3.8 μg/g and values below the threshold were considered as 0 μg/g in the analyses. Subjects were stratified into three groups based on fecal calprotectin levels and commonly used thresholds^56^: low (≤ 50 μg/g), moderate (> 50–100 μg/g) and high (> 100 μg/g). The highest 1% (7 individuals, > 400 μg/g) were excluded as outliers as potential infections and/or undiagnosed IBD might underlie such extreme values.

The gut microbiome was determined using 16S rRNA gene sequencing of stool samples as described in detail previously^55^. Briefly, after DNA extraction, the V3–V4 regions of the bacterial 16S ribosomal RNA, gene were amplified with polymerase chain reactions and sequenced on three separate runs on a MiSeq (Illumina, San Diego, CA; v3 600 cycle kit, forward/reverse read length 328/278 bases)^57^. The raw sequence data was cleaned for primers and low-quality sequences. Alignment of sequences to a reference database (SILVA, v132), chimera removal, taxonomic classification (reference: Ribosomal Database Project (RDP), v16, 16S rRNA reference (PDS)), and operational taxonomic unit (OTU) clustering were run with mothur (v1.40.0)^58^. In addition to differential abundance of microbial taxa, we determined microbial alpha-diversity (Shannon index and inverse Simpson index; R package: phyloseq)^59^ and beta-diversity (Bray–Curtis dissimilarity; R package: vegan).

A panel of 92 inflammatory markers was measured from the serum samples using a Proximity Extension Assay (PEA) assay (Target 96 Inflammation Panel; Olink). Briefly, serum samples were thawed and 20 μl of each sample were used for analysis. In the assay a pair of oligonucleotide-labeled antibodies (PEA-probes) bind to the target protein in the serum sample. When the two probes are in close proximity, a new PCR target sequence is formed by a proximity-dependent DNA polymerization event. This complex is subsequently detected and quantified using standard real-time qPCR. The data quality was monitored throughout the runs using the same inhouse control serum in every running batch. The relative concentration data was normalized and reported on a log2 scale in arbitrary units.

Serum metabolites were relatively quantified using targeted metabolomics of 460 metabolites (135 detected) based on validated^60^ liquid chromatography–mass spectrometry (LC–MS) methods. Briefly, metabolites were extracted from 100 µl serum with 400 µl of extraction solution (ACN:MeOH:MQ) centrifuged. Supernatants were taken to phospholipid removal (Phree Phospholipid removal 96 well plate, Phenomenex) in vacuum. Filtered samples were evaporated and dried and finally reconstituted into 40 µl of extraction solution (ACN:MeOH:MQ). For analysis, 2 µl of LC–MS samples were injected to Vanquish UHPLC (Thermo Fischer Scientific) coupled with Q-Exactive Orbitrap mass spectrometer (Thermo Fischer Scientific). Separation was performed at flow rate of 0.100 ml/minutes with SeQuant ZIC-pHILIC column (Merck) having gradient within 24 min (from 80% B to 20% B), using acetonitrile as mobile phase B and 20 mM ammonium hydrogen carbonate, pH 9.4, as mobile phase A. The MS was equipped with a heated electrospray ionization source using polarity switching and resolution for 70,000. Instrument control was operated with the Xcalibur 4.1.31.9 software (Thermo Fischer Scientific) and the TraceFinder 4.1 software (Thermo Fischer Scientific) was used for the peak integration for standardized peaks for 460 metabolites (MSMLS-1EA, Merck). The data quality was monitored throughout the runs using both a pooled Quality Control (QC) sample prepared by pooling 5 µl from each study sample and an inhouse control serum sample interspersed throughout the run as every 10th sample. Same quality control LC–MS sample and a freshly prepared inhouse QC serum sample were run with each batch. The data was pre-filtered for metabolites having poor chromatograph, intensity variation > 20% RSD in QC sample or carryover. The intensity peak areas of batches data were normalized by probabilistic quotient normalization to a reference inhouse QC sample using MetaboAnalyst 5.0 (www.metaboanalyst.ca). Missing variables were imputed by replacing with 1/5 of the min positive value for each variable and removed with 20% missing value threshold. Data was normalized with log10-transformation and auto-scaled (mean-centered and divided by standard deviation of each variable).

### Demographic, physiological and lifestyle variables

A comprehensive list of demographic, physiological, lifestyle/diet and clinical variables is provided in the Supplementary material. Key variables included age, sex, BMI (kg/m^2^), IBS, functional bloating and constipation (Rome-III criteria), physical inactivity (hours of physical activity per week) and wide range of dietary variables and medications (e.g., cardiovascular, neurological/psychiatric, metabolic, gastrointestinal and anti-inflammatory drugs) across a wide range of indications.

### Health-related and clinical variables

The current subjective health status was self-rated using a horizontal visual analog self-report scale (as part of the EQ-5D-5L) from 0 to 100, where 0 is worst imaginable health and 100 is best imaginable health^61^. The prevalent and incident diagnosis of PD (i.e., at or before wave 4 and at wave 5 or 6, respectively) was made by a neurologist. The prevalent and incident diagnosis of mild cognitive impairment and AD was made by a psychiatrist based on cognitive testing and additional clinical interviews. The severity of acute depression was assessed using the Beck’s Depression Inventory-II (BDI-II sum score). Moreover, depression was indicated based on lifetime diagnosis of depression and/or acute depression (BDI-II > 13). Additionally, the medical history of diseases of interest included (cardio-)vascular diseases (e.g., heart failure, heart attack, stroke, hypertension and thrombosis) and metabolic diseases and parameters (e.g., diabetes type II, hyperlipidemia and high cholesterol) as well as inflammatory diseases (arthritis, arthrosis, IBS) was self-reported by participants.

### Statistical analyses

To identify potential confounders of fecal calprotectin group differences in microbial, serum marker and clinical measures, we investigated 93 variables across demographic, health-related, medication-related, and lifestyle domains (see Supplementary material). Variables were first tested individually for statistical trends (p < 0.1) of fecal calprotectin group differences using Wilcoxon or Fisher’s exact tests. Potential confounders differing (p < 0.05) between the selected fecal calprotectin groups while additionally accounting for age, sex and BMI in logistic regressions were retained as covariates for the respective pairwise fecal calprotectin group comparisons in subsequent analyses.

For analysis of differential abundance in microbial taxa between fecal calprotectin groups, we utilized the R packages phyloseq (1.42.0) and DESeq2 (1.38.3), which models sequence count data using a negative binomial distribution within a generalized linear model. Only taxa present in > 10% of samples were included in the analyses. In addition to potential confounders of calprotectin, further variables associated with microbial composition were considered as covariates comprising intake of antibiotics, antidiabetic drugs, urate lowering drugs, any antihypertensive drugs, physical inactivity, and constipation. Wald tests were used for testing fecal calprotectin group differences in microbial abundance, while adjusting for multiple testing using false discovery rate (FDR; Benjamini–Hochberg method) corrections accounting for the number of different taxa tested. Linear multiple regressions were used to test for calprotectin group differences in α-diversity, and permutational multivariate analyses of variance (PERMANOVA; vegan package (2.6–4)) for differences in Bray–Curtis distances related to β-diversity.

Functional metagenomes were inferred from 16S rRNA gene sequencing data using PICRUSt2 (Phylogenetic Investigation of Communities by Reconstruction of Unobserved States; v2.5.2)^62^. Briefly, first OTU sequences were aligned to a reference^63^ and to pathway classifications using the MetaCyc database. The resulting pathway abundance data (with > 10% prevalence) were employed in a differential abundance analysis of fecal calprotectin groups (accounting for confounders as covariates) using DESeq2 with FDR correction for the number of pathways.

Differences in serum inflammation markers and serum metabolites between calprotectin groups were tested using multiple regressions while accounting for potential confounders. P-values were FDR corrected for multiple testing considering tests of 92 inflammatory markers and 135 metabolites, respectively.

Clinical variables were tested for association with fecal calprotectin groups using Wilcoxon or Fisher’s exact tests, and variables with a fecal calprotectin group difference with p < 0.1 were furthermore tested in a multiple or logistic regression additionally containing age, sex and BMI as covariates (the latter was omitted for metabolic diseases).

Mediation analysis was performed using the R package LDM (6.0.1)^64^ for each of the pair-wise group comparisons while accounting for covariates. Fecal calprotectin group was used as the exposure variable for the mediation analyses, while the genera identified to be significantly differentially abundant between fecal calprotectin groups served as mediators, and the serum markers significantly differing between fecal calprotectin groups (indoxyl sulfate, IL-17C and CCL19) were used as separate outcome variables. Thus, three mediation analyses were conducted per group comparison. The linear decomposition model (LDM) is based on linear models of regressing individual taxon data on the sequentially orthogonalized covariates while controlling for confounding variables. Mediation effects were tested for global mediation effects of 12 taxa as well as at the individual taxon level. The inference of associations relies on permutation to avoid making parametric assumptions regarding the distribution of the microbiome data. Omni3 results are reported, which combine findings from analyses conducted on frequency, arcsine-root-transformed, and presence-absence scales. This approach optimizes power across various scenarios, as relative abundance is more accurate for abundant taxa while presence-absence is better suited for rare taxa. P-values were adjusted for multiple testing using the algorithm for false discovery rate (FDR) modulated sequential Monte Carlo (MC) multiple hypothesis testing within the LDM package.

TREND study data were collected and managed using REDCap electronic data capture tools hosted at the University of Tübingen. All statistical analyses were performed with software R version 4.2.2^65^ and figures were generated using ggplot2 (3.4.2).

### Supplementary Information

Supplementary Figures.

Supplementary Tables.

## Data Availability

The TREND cohort data can be shared upon justified request. The raw sequence data of the gut microbiome is available on the ENA server using the Accession Number PRJEB32920.

## Abbreviations

AD: Alzheimer’s disease
BDI-II: Beck’s Depression Inventory-II
BMI: Body mass index
CVD: Cardiovascular disease
EQ-5D-5L: EuroQol-5 Dimensions 5-Level version
FDR: False discovery rate
IBD: Inflammatory bowel disease
IBS: Irritable bowel syndrome
MDS-UPDRS-III: Movement Disorder Society-Unified Parkinson’s Disease Rating Scale Part III
NSAIDs: Nonsteroidal anti-inflammatory drugs
OTU: Operational taxonomic unit
PD: Parkinson’s disease
PICRUSt2: Phylogenetic Investigation of Communities by Reconstruction of Unobserved States 2.0
PERMANOVA: Permutational multivariate analysis of variance
PPI: Proton-pump inhibitor
SCFA: Short-chain fatty acid
SD: Standard deviation
TREND: Tübingen evaluation of Risk factors for Early detection of NeuroDegeneration

## References

[1] Jukic A, Bakiri L, Wagner EF, Tilg H, Adolph TE (2021). Calprotectin: From biomarker to biological function. Gut.

[2] Mendall MA, Chan D, Patel R, Kumar D (2016). Faecal calprotectin: Factors affecting levels and its potential role as a surrogate marker for risk of development of Crohn’s Disease. BMC Gastroenterol..

[3] Lundgren D, Eklöf V, Palmqvist R, Hultdin J, Karling P (2019). Proton pump inhibitor use is associated with elevated faecal calprotectin levels. A cross-sectional study on subjects referred for colonoscopy. Scand. J. Gastroenterol..

[4] Agirman G, Yu KB, Hsiao EY (2021). Signaling inflammation across the gut-brain axis. Science.

[5] Franceschi C, Garagnani P, Parini P, Giuliani C, Santoro A (2018). Inflammaging: A new immune–metabolic viewpoint for age-related diseases. Nat. Rev. Endocrinol..

[6] Mulak A, Koszewicz M, Panek-Jeziorna M, Koziorowska-Gawron E, Budrewicz S (2019). Fecal calprotectin as a marker of the gut immune system activation is elevated in Parkinson’s disease. Front. Neurosci..

[7] Aho VTE (2021). Relationships of gut microbiota, short-chain fatty acids, inflammation, and the gut barrier in Parkinson’s disease. Mol. Neurodegener..

[8] Leblhuber F, Geisler S, Steiner K, Fuchs D, Schütz B (2015). Elevated fecal calprotectin in patients with Alzheimer’s dementia indicates leaky gut. J. Neural Transm..

[9] Heston MB (2023). Gut inflammation associated with age and Alzheimer’s disease pathology: A human cohort study. Sci. Rep..

[10] Franzosa EA (2019). Gut microbiome structure and metabolic activity in inflammatory bowel disease. Nat. Microbiol..

[11] Forbes JD (2018). A comparative study of the gut microbiota in immune-mediated inflammatory diseases—Does a common dysbiosis exist?. Microbiome.

[12] Vogt NM (2017). Gut microbiome alterations in Alzheimer’s disease. Sci. Rep..

[13] Wallen ZD (2022). Metagenomics of Parkinson’s disease implicates the gut microbiome in multiple disease mechanisms. Nat. Commun..

[14] Pinart M (2022). Gut microbiome composition in obese and non-obese persons: A systematic review and meta-analysis. Nutrients.

[15] Jie Z (2017). The gut microbiome in atherosclerotic cardiovascular disease. Nat. Commun..

[16] Pascal V (2017). A microbial signature for Crohn’s disease. Gut.

[17] Fusco W (2023). Short-chain fatty-acid producing bacteria: Key components of the human gut microbiota. Nutrients.

[18] Silva YP, Bernardi A, Frozza RL (2020). The role of short-chain fatty acids from gut microbiota in gut-brain communication. Front. Endocrinol..

[19] Heinzel S (2019). Update of the MDS research criteria for prodromal Parkinson’s disease. Mov. Disord..

[20] Aldars-García L, Chaparro M, Gisbert JP (2021). Systematic review: The gut microbiome and its potential clinical application in inflammatory bowel disease. Microorganisms.

[21] Zeng MY, Inohara N, Nuñez G (2017). Mechanisms of inflammation-driven bacterial dysbiosis in the gut. Mucosal Immunol.

[22] Mohr AE, Crawford M, Jasbi P, Fessler S, Sweazea KL (2022). Lipopolysaccharide and the gut microbiota: Considering structural variation. FEBS Lett..

[23] Heidarian F, Alebouyeh M, Shahrokh S, Balaii H, Zali MR (2019). Altered fecal bacterial composition correlates with disease activity in inflammatory bowel disease and the extent of IL8 induction. Curr. Res. Transl. Med..

[24] Schirmer M (2018). Compositional and temporal changes in the gut microbiome of pediatric ulcerative colitis patients are linked to disease course. Cell Host Microbe.

[25] Sayols-Baixeras S (2023). Streptococcus species abundance in the gut is linked to subclinical coronary atherosclerosis in 8973 participants from the SCAPIS cohort. Circulation.

[26] Rastogi S, Singh A (2022). Gut microbiome and human health: Exploring how the probiotic genus Lactobacillus modulate immune responses. Front. Pharmacol..

[27] Jemimah S, Chabib CMM, Hadjileontiadis L, AlShehhi A (2023). Gut microbiome dysbiosis in Alzheimer’s disease and mild cognitive impairment: A systematic review and meta-analysis. PLoS ONE.

[28] Li C, Peng K, Xiao S, Long Y, Yu Q (2023). The role of Lactobacillus in inflammatory bowel disease: From actualities to prospects. Cell Death Discov..

[29] Rossi F, Amadoro C, Colavita G (2019). Members of the lactobacillus genus complex (LGC) as opportunistic pathogens: A review. Microorganisms.

[30] Serrano-Gómez G (2021). Dysbiosis and relapse-related microbiome in inflammatory bowel disease: A shotgun metagenomic approach. Comput. Struct. Biotechnol. J..

[31] Bhatti MA, Frank MO (2000). Veillonella parvula meningitis: Case report and review of Veillonella infections. Clin. Infect. Dis..

[32] Hoare A (2021). A cross-species interaction with a symbiotic commensal enables cell-density-dependent growth and in vivo virulence of an oral pathogen. ISME J..

[33] Weersma RK, Zhernakova A, Fu J (2020). Interaction between drugs and the gut microbiome. Gut.

[34] Witkowski M, Weeks TL, Hazen SL (2020). Gut microbiota and cardiovascular Disease. Circ. Res.

[35] Albillos A, de Gottardi A, Rescigno M (2020). The gut-liver axis in liver disease: Pathophysiological basis for therapy. J. Hepatol..

[36] Lee YS (2018). Microbiota-derived lactate accelerates intestinal stem-cell-mediated epithelial development. Cell Host Microbe.

[37] Bartolomaeus H (2019). Short-chain fatty acid propionate protects from hypertensive cardiovascular damage. Circulation.

[38] Hoyles L (2018). Microbiome–host systems interactions: Protective effects of propionate upon the blood–brain barrier. Microbiome.

[39] McGeachy MJ, Cua DJ, Gaffen SL (2019). The IL-17 family of cytokines in health and disease. Immunity.

[40] Friedrich M, Diegelmann J, Schauber J, Auernhammer CJ, Brand S (2015). Intestinal neuroendocrine cells and goblet cells are mediators of IL-17A-amplified epithelial IL-17C production in human inflammatory bowel disease. Mucosal Immunol..

[41] Westermann J (2007). CCL19 (ELC) as an adjuvant for DNA vaccination: Induction of a TH1-type T-cell response and enhancement of antitumor immunity. Cancer Gene Ther..

[42] Leong SC, Sirich TL (2016). Indoxyl sulfate-review of toxicity and therapeutic strategies. Toxins.

[43] Paeslack N (2022). Microbiota-derived tryptophan metabolites in vascular inflammation and cardiovascular disease. Amino Acids.

[44] Vanholder R, Schepers E, Pletinck A, Nagler EV, Glorieux G (2014). The uremic toxicity of indoxyl sulfate and p-cresyl sulfate: A systematic review. J. Am. Soc. Nephrol..

[45] Huang Y (2020). Indoxyl sulfate induces intestinal barrier injury through IRF1-DRP1 axis-mediated mitophagy impairment. Theranostics.

[46] Yamaguchi K (2022). Indoxyl sulfate activates NLRP3 inflammasome to induce cardiac contractile dysfunction accompanied by myocardial fibrosis and hypertrophy. Cardiovasc. Toxicol..

[47] Ntranos A (2022). Bacterial neurotoxic metabolites in multiple sclerosis cerebrospinal fluid and plasma. Brain.

[48] Kunutsor SK (2018). Plasma calprotectin and risk of cardiovascular disease: Findings from the PREVEND prospective cohort study. Atherosclerosis.

[49] Wang X (2021). Gut microbiota in NSAID enteropathy: New insights from inside. Front. Cell. Infect. Microbiol..

[50] Grand A (2020). Body mass index and calprotectin blood level correlation in healthy children: An individual patient data meta-analysis. J. Clin. Med..

[51] Chan SSM (2022). Obesity is associated with increased risk of Crohn’s disease, but not ulcerative colitis: A pooled analysis of five prospective cohort studies. Clin. Gastroenterol. Hepatol..

[52] Mendall M, Harpsøe MC, Kumar D, Andersson M, Jess T (2018). Relation of body mass index to risk of developing inflammatory bowel disease amongst women in the Danish National Birth Cohort. PLoS ONE.

[53] Mouton AJ, Li X, Hall ME, Hall JE (2020). Obesity, hypertension, and cardiac dysfunction novel roles of immunometabolism in macrophage activation and inflammation. Circ. Res..

[54] Portincasa P (2022). Gut microbiota and short chain fatty acids: Implications in glucose homeostasis. Int. J. Mol. Sci..

[55] Heinzel S (2021). Gut microbiome signatures of risk and prodromal markers of Parkinson disease. Ann. Neurol..

[56] Kim ES (2021). Optimal cutoff level of fecal calprotectin for detecting small bowel inflammation in Crohn’s disease. Gut Liver.

[57] Kozich JJ, Westcott SL, Baxter NT, Highlander SK, Schloss PD (2013). Development of a dual-index sequencing strategy and curation pipeline for analyzing amplicon sequence data on the MiSeq Illumina sequencing platform. Appl. Environ. Microbiol..

[58] Schloss PD (2009). Introducing mothur: Open-source, platform-independent, community-supported software for describing and comparing microbial communities. Appl. Environ. Microbiol..

[59] McMurdie PJ, Holmes S (2013). Phyloseq: An R package for reproducible interactive analysis and graphics of microbiome census data. PLoS ONE.

[60] EATRIS Plus Multi-omics Working Group and Stakeholders (2023). Multi-omics quality assessment in personalized medicine through EATRIS. BioRxiv..

[61] Janssen MF, Pickard AS, Shaw JW (2021). General population normative data for the EQ-5D-3L in the five largest European economies. Eur. J. Health Econ..

[62] Douglas GM (2020). PICRUSt2 for prediction of metagenome functions. Nat. Biotechnol..

[63] Barbera P (2019). EPA-ng: Massively parallel evolutionary placement of genetic sequences. Syst. Biol..

[64] Yue Y, Hu YJ (2022). A new approach to testing mediation of the microbiome at both the community and individual taxon levels. Bioinformatics.

[65] R Core Team. *R: A Language and Environment for Statistical Computing* (2022).

