# Supplementary material for: Elevated fecal calprotectin is associated with gut microbial dysbiosis, altered serum markers and clinical outcomes in older individuals
Source: Sci Rep. 2024 Jun 12;14:13513. doi: 10.1038/s41598-024-63893-0 (PMC11169261; doi:10.1038/s41598-024-63893-0)
Supplement: Supplementary file 1 — Supplementary Figures. [file 41598_2024_63893_MOESM1_ESM.docx]

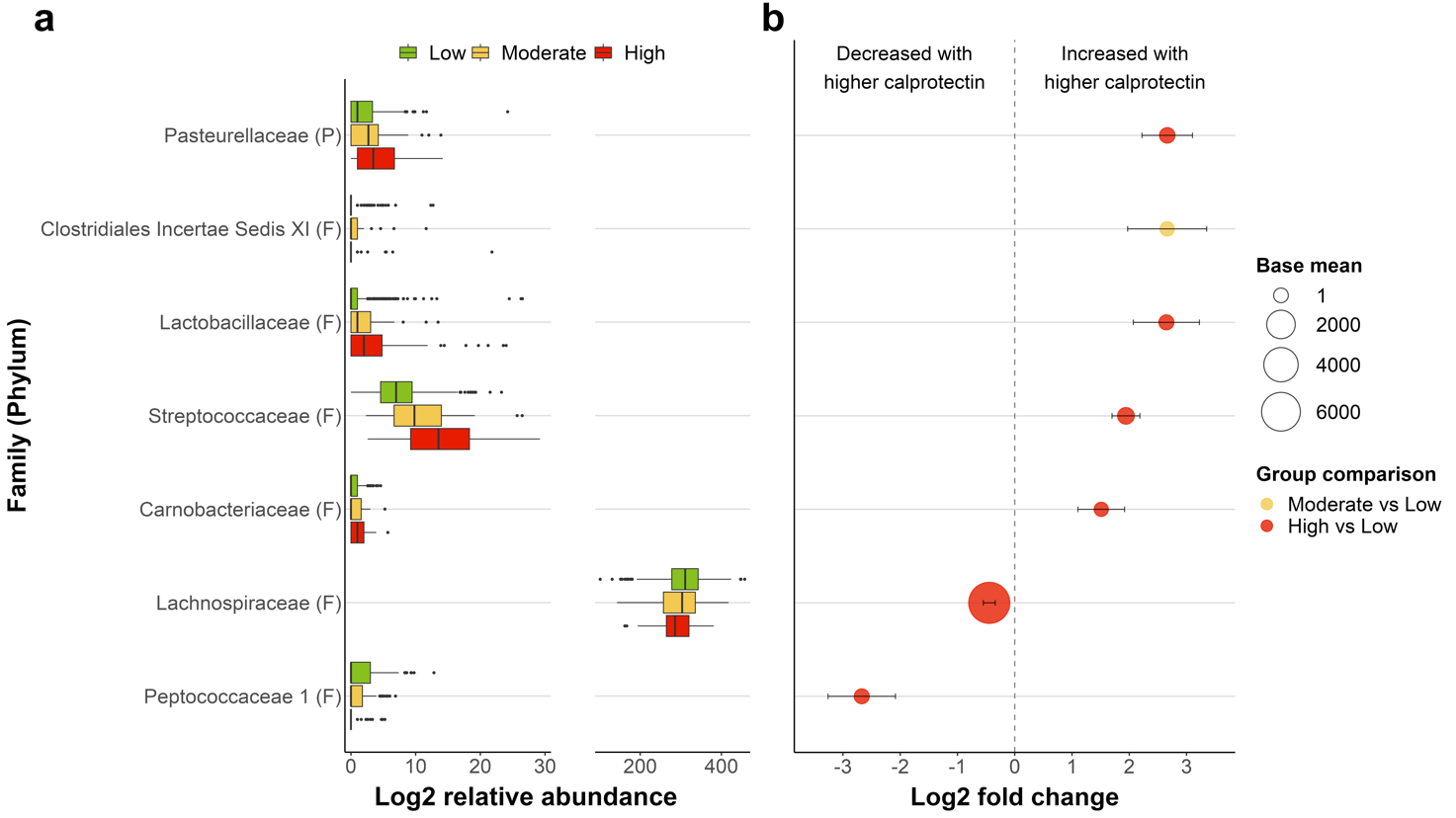


**Supplementary Figure 1.** Family-level differential abundance analysis. (A) Group-wise boxplots of the relative abundance (log2) of families that show significant differences in group contrasts. (B) Significant (FDR-corrected, covariate-adjusted) findings of differential abundance of gut microbial families for comparisons between low (≤50 μg/g) and moderate (>50-100 μg/g) or high (>100 μg/g) calprotectin groups. The size of the markers corresponds to the base mean value, i.e., the mean of normalized counts of all samples, and the markers are colour-coded based on group comparison. Bars indicate the standard error of log2-fold change. The phylum of the families is indicated in brackets. Abbreviations: F, Firmicutes; P, Proteobacteria.


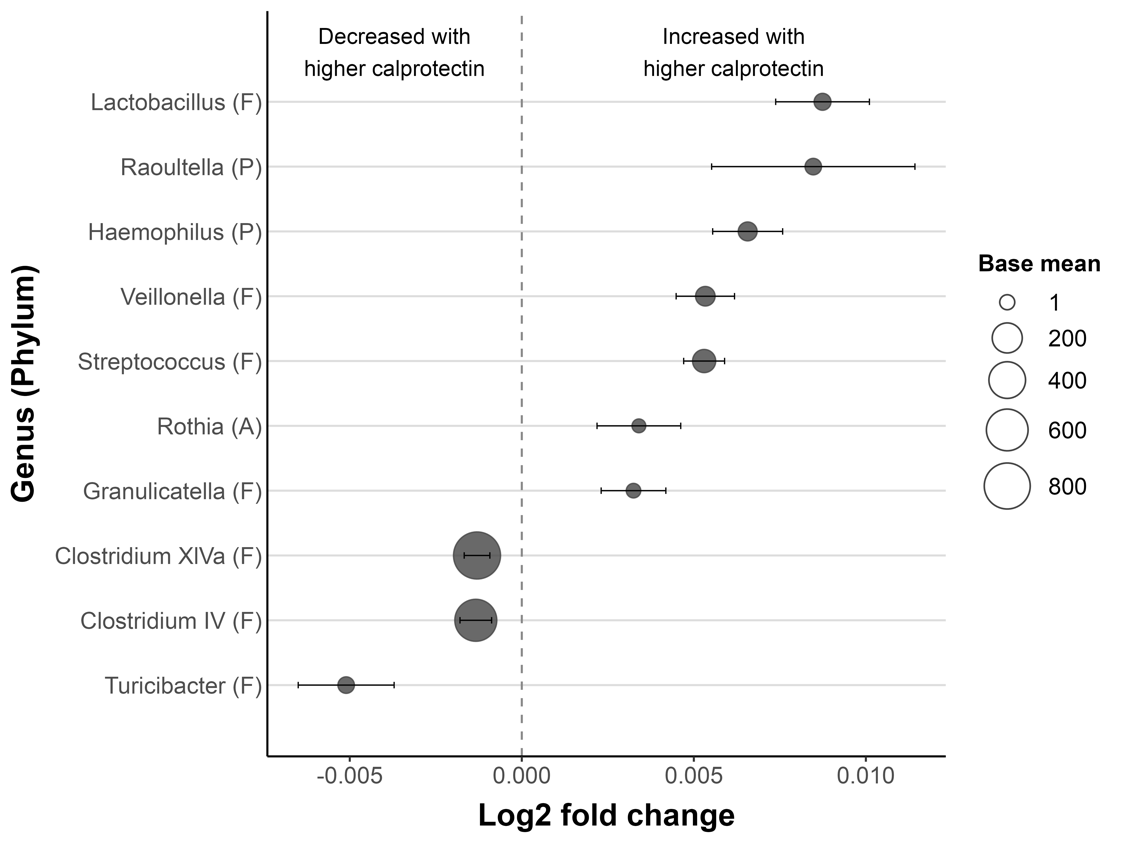


**Supplementary Figure 2.** Genus-level differential abundance analysis using fecal calprotectin as a continuous variable (n=735). The size of the markers corresponds to the base mean value, i.e., the mean of normalized counts of all samples, and bars indicate the standard error of log2-fold change. Phylum abbreviations: F, Firmicutes; P, Proteobacteria; A, Actinobacteria.
